# Sucrose-Sweetened Drinks Reduce the Physical Performance and Increase the Cardiovascular Risk in Physically Active Males

**DOI:** 10.1155/2021/6683657

**Published:** 2021-03-09

**Authors:** Raianne dos Santos Baleeiro, Aparecida Patricia Guimarães, Perciliany Martins de Souza, Rafael da Silva Andrade, Karina Barbosa de Queiroz, Daniel Barbosa Coelho, Emerson Cruz de Oliveira, Lenice Kappes Becker

**Affiliations:** ^1^Health and Nutrition, PPGSN, Research Center in Biological Sciences, Federal University of Ouro Preto, Ouro Preto, Minas Gerais, Brazil; ^2^Health and Nutrition, PPGSN, Physical Education Department, Physical Education School, Federal University of Ouro Preto, Ouro Preto, Minas Gerais, Brazil; ^3^Research Center in Biological Sciences, Physical Education Department, Physical Education School, Federal University of Ouro Preto, Ouro Preto, Minas Gerais, Brazil; ^4^Health and Nutrition, PPGSN, Food Department, Nutrition School, Federal University of Ouro Preto, Ouro Preto, Minas Gerais, Brazil

## Abstract

**Introduction:**

The intake of sugar-sweetened beverages (SSBs) has increased rapidly, but the effects of this habit on health and physical performance are unknown. This study assessed the effect of excessive SSB intake on biochemical, physical performance, and biochemical and cardiovascular parameters of physically active males.

**Methods:**

Seventeen volunteers consumed a placebo drink (Pd; carbohydrate free) and an excessive SSB drink (eSSBd = Pd plus 300 g sucrose). In a blind randomized crossover study, the subjects were assigned to Pd or eSSBd groups for 15 days. After an interval of 7 days, subjects were reassigned to the other condition.

**Results:**

After eSSBd intake, there was an increase in weight (69.34 ± 13.71 vs. 70.62 ± 14.06), body mass index (24.49 ± 4.01 vs. 24.97 ± 4.13), waist circumference (75.33 ± 11.22 vs. 76.79 ± 11.51), VLDL (19.54 ± 9.50 vs. 25.52 ± 11.18), triglycerides (78.94 ± 23.79 vs. 114.77 ± 43.65), and peak systolic blood pressure (178.57 ± 26.56 vs. 200.71 ± 24.64). The cardiorespiratory response to exercise (VO_2_max) (48.15 ± 10.42 vs. 40.98 ± 11.20), peak heart rate (186.64 ± 8.00 vs. 179.64 ± 6.28), total exercise time (15.02 ± 1.57 vs. 14.00 ± 2.18), and mechanical work (15.83 ± 4.53 vs. 13.68 ± 5.67) decreased after eSSBd intake (all values expressed in initial mean ± DP vs. final). The rates of perceived exertion were higher (1.300 vs.1.661 slope and −0.7186 vs. −1.118 *y*-intercept) after eSSBd intake.

**Conclusion:**

The present study shows that 15 days of eSSBd intake may negatively modulate biochemical parameters associated with cardiovascular risk. In addition, this overintake can impair the physical performance and cardiovascular responses to physical exercise.

## 1. Introduction

Over the last 20–30 years, the world population has reduced water intake and the intake of many healthy foods, such as legumes, fruits, and vegetables. On the contrary, the intake of sugar-sweetened beverages (SSBs) and ultraprocessed foods has increased rapidly [1].

Some studies have shown that excess carbohydrate intake can impair cardiovascular health [2, 3]. Another study conducted in adults showed that a high amount of SSB induced an increase in blood pressure (BP) and incidence of hypertension, independent of body weight [4]. A study in animal models showed that excessive fructose intake activates the autonomic nervous system (ANS) and thereby increases BP [5].

An excessive intake of SSB is also a risk factor for cardiovascular diseases through biochemical alterations as SSB stimulates lipogenesis and increases triglycerides (TG) and total serum cholesterol levels [6, 7].

A recent study has shown that 14 days of a sucrose-supplemented diet attenuated vascular function in young healthy individuals during leg exercise. This impairment resulted from reduced activity of endothelial nitric oxide (NO) synthase. It is well established that NO contributes to the physical performance through increased perfusion of oxygen and nutrients; therefore, SSB can decrease the exercise performance via a decrease in vascular flux [8].

The relationship between exercise and excessive intake of SSB has been investigated, and studies have shown that exercise may prevent transient SSB-mediated microvascular endothelial dysfunction [9]. In addition, a study using transmission electron microscopy found a greater amount of abnormal mitochondria in gastrocnemius muscles of rats treated with diets rich in simple carbohydrates, and this effect was controlled by exercise training [10]. On the contrary, our group showed that exercise in young animals (21 days old) did not prevent an increase in serum TG levels caused by a high-sugar diet [11].

Excess sugar intake induced a decrease in physical activity in rats [12], and there is an association in humans between sedentary habits and SSB intake [13]. One systematic review noted that the intake of SSB was associated with low aerobic performance [14].

Physical performance is related to carbohydrate ingestion, and it was noted that there is metabolic flexibility; that is, the body prioritizes the use of carbohydrates as the predominant substrate during exercise [15, 16]. In an intervention with higher amounts of carbohydrates before and during exercise, it was found that an increase in exogenous glucose intake leads to a significant reduction in lipid oxidation, but not in carbohydrates, and leads to changes in the expression of proteins related to energy metabolism [15].

Furthermore, carbohydrate intake has long been understood to be important for good physical performance [17]. Nevertheless, there are important gaps to be examined, such as the relationship between excessive carbohydrate intake and physical performance. Studies that investigated the relationship between carbohydrate intake, lipid oxidation, and physical exercise show that a higher carbohydrate intake immediately after exercise induces an increase in plasma levels of postprandial insulin, inhibits lipogenesis, and contributes to systemic lipid oxidation decrease [18, 19].

Considering the effects of excessive SSB intake on the cardiovascular system and the impact on physical performance, the present study has two hypotheses: excessive SSB intake worsens biochemical and cardiovascular parameters after exercise as well as reduces the physical performance.

## 2. Materials and Methods

### 2.1. Participants

The volunteers were seventeen active healthy males with a mean age of 24.92 ± 4.34 years, mean weight of 75.62 ± 11.01 kg, mean height of 176 ± 0.11 cm, and mean body mass index (BMI) of 24.59 ± 3.98. The inclusion criteria were as follows: practicing physical activity regularly at least 3 times a week and being available to visit the laboratory for 4 morning meetings. We excluded volunteers with diabetes mellitus, hypertension, physical injury, or sedentary status. All participants signed a consent form. The study was approved by the ethics committee for studies with humans of the Federal University of Ouro Preto under approval number 56317816.3.0000.5150.

### 2.2. Study Design

**Figure 1:**
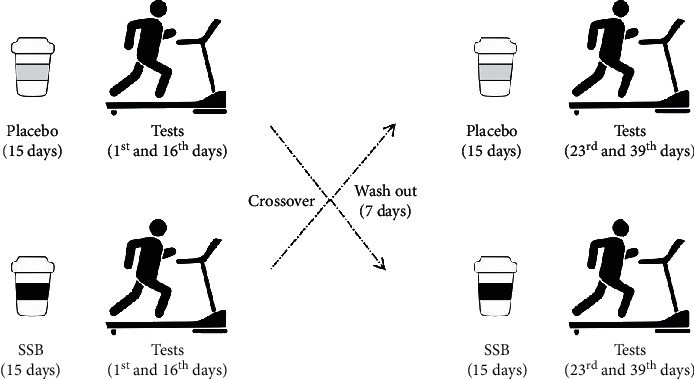
Experimental design.

A blind crossover randomized study was performed. Subjects were randomly assigned a number using a website (https://www.random.org/lists/) and assigned into one of two experimental conditions: placebo (SP) or sucrose (SS). Each treatment was performed for 15 days, followed by 7 days of interval between conditions (shown in Figure 1). In each experimental period, placebo (PA) and sucrose (SU) beverages were offered. PA consisted of flavored unsweetened juice (Clight® São Paulo, SP, Brazil; composition: 0 g carbohydrates), and SU was composed of the same drink with 300 g of sucrose and citric extract added. Both drinks were consumed daily and had the volume supplemented with water to 1.5 L. The drink was consumed in 4 portions during the day according to voluntary demand. All drinks were prepared by the same researcher, and the volunteers did not have knowledge of which drink they were ingesting. The sample size was determined using heart rate (HR) differences with a power of 0.90, assuming an effect size of 0.8 and type I error of 0.05 (alpha). Volunteers were recruited through the social network of the Federal University of Ouro Preto between 2018 and 2019.

Subjects were instructed to maintain a balanced diet (55% carbohydrate, 30% fat, and 15% protein), perform physical activities normally, and avoid training modifications during the study. The drinks were delivered to volunteers daily for intake certification and routine guidelines. In order to avoid that the volunteers spent many days without contact with the researchers, the washout was fixed at seven days, so it was intended to avoid significant changes in the dietary routine and/or physical activity levels that could interfere in the research results.

On the day of each experimental condition, the volunteers visited our laboratory in the morning. After 8 h of fasting, blood samples were collected in 9 mL tubes without the anticoagulant and centrifuged at 1080 G. The serum was fractionated into 1 mL aliquots across 3 microtubes and stored at −20°C.

### 2.3. Biochemical Analysis

The concentrations of fasting blood glucose, total cholesterol, high-density lipoprotein (HDL), very-low-density lipoprotein (VLDL), and TG were assessed using the Bioclin® kit (Belo Horizonte; Minas Gerais, Brazil). All blood measurements were performed in triplicate.

### 2.4. Anthropometric and Resting Cardiovascular Measurements

The biometric parameters were measured: weight, height, and fat percentage [20]. Systolic blood pressure (SBP) and diastolic blood pressure (DBP) were measured using an aneroid sphygmomanometer and a stethoscope. Resting heart rate (HR) was assessed using a Polar® RS800 (Kempele, Oulu, Finland) HR monitor. Thereafter, a standardized breakfast was given to the volunteers prior to the physical performance test.

### 2.5. Assessment of the Physical Performance and Cardiovascular Response

In each of the laboratory visits, the volunteers were submitted to the Bruce incremental treadmill protocol until voluntary exhaustion [21]. VO_2_max was directly measured using a VO2000® analyzer (MGC Diagnostics; St. Paul, MN). Total exercise time (TET), cardiovascular response, SBP, and DBP throughout the test were assessed. Ratings of perceived exertion (RPE) were checked at each stage using the Borg scale [22].

### 2.6. Statistical Analysis

Data normality was tested using the D'Agostino–Pearson test. Data that were normally distributed were analyzed with a two-way repeated measure ANOVA followed by Bonferroni's multiple comparisons test. A regression was used when indicated. In results, data were expressed as mean ± standard deviation (SD), and the significance level was set at *p* < 0.05.

## 3. Results

**Figure 2:**
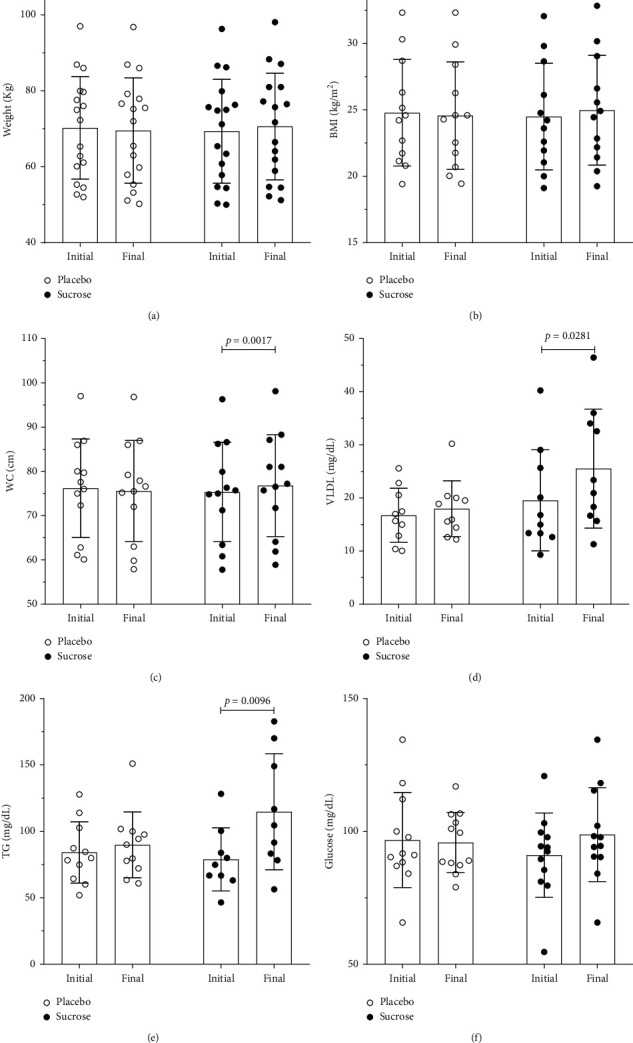
Anthropometric, body composition, and metabolic parameters. (a) Weight, 95% CI of the difference between initial vs. final (−0.7009 to 0.1368), no differences, 95% CI of the difference between placebo vs. sucrose (−9.710 to 9.534), *p*=0.0002 (Bonferroni's multiple comparisons test), *n* = 17. (b) BMI = body mass index, 95% CI of the difference between initial vs. final (−0.3136 to 0.04690), no differences, 95% CI of the difference between placebo vs. sucrose −3.481 to 3.368), *p*=0.0016 (Bonferroni's multiple comparisons test), *n* = 12. (c) WC = waist circumference, 95% CI of the difference between initial vs. final (−0.9632 to 0.1474), no differences, 95% CI of the difference between placebo vs. sucrose (−9.742 to 9.393), *p*=0.0017 (Bonferroni's multiple comparisons test), *n* = 12. (d) VLDL = very-low-density lipoproteins, 95% CI of the difference between initial vs. final (−6.872 to −0.3415), no differences, 95% CI of the difference between placebo vs. sucrose (−12.15 to 1.796), *p*=0.0281 (Bonferroni's multiple comparisons test), *n* = 10. (e) TG = triglycerides, 95% CI of the difference between initial vs. final (−36.55 to −4.970), no differences, 95% CI of the difference between placebo vs. sucrose (−32.73 to 13.07), *p*=0.0096 (Bonferroni's multiple comparisons test), *n* = 10. (f) Glucose, 95% CI of the difference between initial vs. final (−11.76 to 4.957), no differences, 95% CI of the difference between placebo vs. sucrose (−9.202 to 11.93), no differences (Bonferroni's multiple comparisons test), *n* = 12; two-way repeated measure ANOVA followed by Bonferroni's multiple comparisons test.

**Figure 3:**
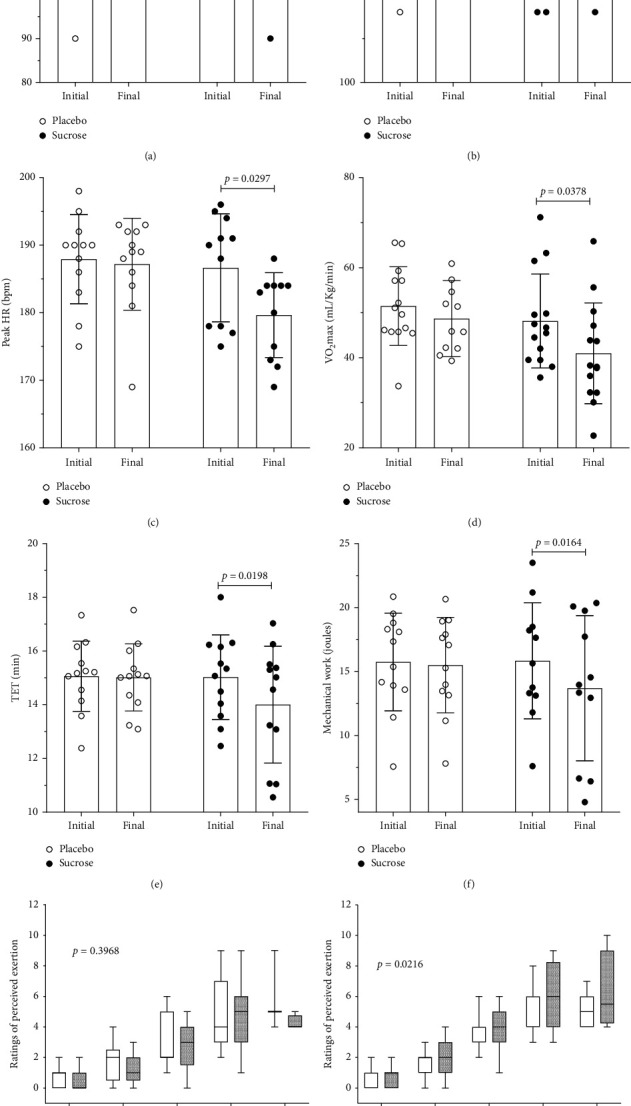
Physiological and performance parameters. (a) Rest systolic blood pressure (SBP), 95% CI of the difference between initial vs. final (−2.747 to 4.081), no differences, 95% CI of the difference between placebo vs. sucrose (−9.432 to 1.432), no difference (Bonferroni's multiple comparisons test), *n* = 15. (b) Peak systolic blood pressure (SBP), 95% CI of the difference between initial vs. final (−22.90 to 2.900), no differences, 95% CI of the difference between placebo vs. sucrose (−21.05 to 11.05), *p*=0.0386 (Bonferroni's multiple comparisons test), *n* = 14. (c) Peak heart hate (HR), 95% CI of the difference between initial vs. final (0.07900 to 7.671), no differences, 95% CI of the difference between placebo vs. sucrose (−0.2743 to 9.085), *p*=0.0297 (Bonferroni's multiple comparisons test), *n* = 11. (d) VO_2_max, 95% CI of the difference between initial vs. final (0.07900 to 7.671), no differences, 95% CI of the difference between placebo vs. sucrose (−0.2743 to 9.085), *p*=0.0378 (Bonferroni's multiple comparisons test), *n* = 14. (e) Total exercise time (TET), 95% CI of the difference between initial vs. final (0.002003 to 1.066), no differences, 95% CI of the difference between placebo vs. sucrose (−0.7387 to 1.787), *p*=0.0198 (Bonferroni's multiple comparisons test), *n* = 12. (f) Mechanical work, 95% CI of the difference between initial vs. final (0.1359 to 2.260), no differences, 95% CI of the difference between placebo vs. sucrose (−2.878 to 4.599), *p*=0.0164 (Bonferroni's multiple comparisons test), *n* = 11; two-way repeated measure ANOVA followed by Bonferroni's multiple comparisons test (a to f). (g) Ratings of perceived exertion; no differences between the two conditions, placebo and sucrose, during the performance of the physical test (initial) (*F* = 0.9325; *p*=0.0386), box and whisker (min to max). (h) Ratings of perceived exertion; there was a significant difference between the two conditions, placebo and sucrose, during the performance of the physical test (final) (*F* = 3.980; *p*=0.0216), box and whisker (min to max). Therefore, sucrose ingestion increases rates of perception exertion. Linear regression (g and h), null hypothesis = one curve for all datasets, and alternative hypothesis = different curves for each dataset.

After 15 days of placebo intake, we did not observe differences in body weight (shown in Figure 2(a)), body mass index (shown in Figure 2(b)), waist circumference (shown in Figure 2(c)), VLDL (shown in Figure 2(d)), triglycerides (shown in Figure 2(e)), and peak systolic blood pressure (shown in Figure 3(b)) and in other parameters not shown in the figures. However, after 15 days of excessive sucrose intake, these same individuals presented an increase in body weight (shown in Figure 2(a)), body mass index (shown in Figure 2(b)), waist circumference (shown in Figure 2(c)), VLDL (shown in Figure 2(d)), triglycerides (shown in Figure 2(e)), and peak systolic blood pressure (shown in Figure 3(b)).

After excessive sucrose intake, we did not observe any differences in glucose (shown in Figure 2(f)) and rest systolic blood pressure (shown in Figure 3(a)). However, there were decreases in peak heart rate (shown in Figure 3(c)), cardiorespiratory response to exercise (VO_2_max) (shown in Figure 3(d)), total exercise time (shown in Figure 3(e)), and mechanical work (shown in Figure 3(f)). Another parameter influenced by excessive sucrose intake was the rates of perceived exertion. At the beginning of the experiment, the two curves were identical (shown in Figure 3(g)), but at the end, there was a significant difference (shown in Figure 3(h)). Therefore, sucrose ingestion increases rates of perception exertion.

## 4. Discussion

The present study evaluated the effect of excessive sucrose intake for 15 days in active healthy young males to study anthropometric parameters, body composition, biochemical parameters related to cardiovascular risk, physical performance, and cardiorespiratory response to exercise.

Some of the analyzed parameters were altered by excessive SSB intake; body weight, body mass index, waist circumference, VLDL, triglycerides, and peak systolic blood pressure were increased when excessive SSB was administered. The physical performance was impaired, and the maximal VO_2_max, total time to exhaustion, and mechanical work decreased after sucrose intake. The biochemical parameters related to the cardiovascular risk as well as TG and VLDL were decreased by excessive SSB intake.

The differences observed after the interventions could be explained by changes in diet. In both interventions, the volunteers drank 1.5 L per day, and it is possible that there was a compensatory response. It has been demonstrated that physiological changes occur day by day to different extents for determined periods when energy intake and expenditure are altered by exercise or diet composition in humans [23]. Studies that manipulated dietary fat and energy density show that increases in a diet's energy density by adding fat appear to lead to little or no change in food intake. These effects are apparent over time periods ranging from 1 day [24] to 2 weeks [25]. Additionally, another experiment demonstrated that metabolic and endocrine changes could be observed 24 hours after high-sucrose intake [26].

Body weight increases after excessive SSB intake accompanied by an increase in BMI (shown in Figure 2). Our results corroborate previous studies that showed a positive association between SSB intake and weight increase [2, 27]. The increase in BMI observed in the present study is also in agreement with findings from other studies [28–30].

Physical performance was impaired after excessive SSB intake. Maximal VO_2_max, which represents cardiorespiratory response, decreases, accompanied by a reduction in TET and mechanical work. VO_2_max is dependent on several physical parameters, including body composition [31, 32]. Excessive SSB intake increased body weight and impaired BMI, which has an important impact on oxygen intake. The decrease in TET to exhaustion and mechanical work showed that excessive SSB intake reduced the physical performance. In addition, RPE was higher after sucrose intake.

The mechanisms by which physical performance is reduced and RPE is increased still need to be elucidated. In adolescents, SSB intake is associated with mental and physical fatigue [33]. Similarly, rats that consumed fructose had reduced physical activity levels [12].

BP tends to change in response to excessive intake of simple carbohydrates [2, 34]. The increase in BP may have been caused by greater vascular stiffness caused by excessive consumption of sucrose. To support this hypothesis, we cite a previous study in which a negative effect of excessive fructose consumption, contained in industrialized products, was observed on arterial aging [35]. Another factor that may have contributed to the increase in BP is an increase in serum uric acid levels. It is already known that, in diets with excessive fructose consumption, there is an increase in uric acid levels, which is already a risk factor for high blood pressure [36]. The sum of factors (increased vascular stiffness and increased serum uric acid levels) also forms another hypothesis for the increase in BP. Our data show an increase in SBP in the effort peak. The effort peak of HR decreased after excessive SSB intake, probably because the HR response accompanied the impairment in VO_2_max.

One hypothesis for physical performance reduction after excessive SSB intake can be due to a decrease in NO bioavailability with consequent vasodilation reduction, impairing the nutrient flux. It has been shown that vascular function may be worsened by high sugar levels, in part due to endothelial NO synthase alterations [8]. Another hypothesis is a decrease in the autonomic function that could be accessed exploring the heart rate variability (HRV). We could not investigate the HRV, so this is one of the study limitations.

There were also alterations of the lipid profile, with a significant increase in TG and VLDL. It is known that diets with high levels of simple carbohydrates have high potential in the lipid synthesis pathway, which changes the concentration of total serum cholesterol, TG, and VLDL [35, 36]. The amount of simple carbohydrate intake used in this study was sufficient to alter the lipid profile significantly.

Carbohydrate intake during exercise (45 g) also induces a decrease in lipid oxidation [37]. Hence, it is assumed that the decrease in the physical performance is due to the reduction of lipid oxidation capacity by a reduction of enzymatic activity, possibly at the mitochondrial level [38, 39]. A recent study [10] showed through transmission electron microscopy that rats trained and treated with diets rich in simple carbohydrates presented greater amounts of abnormal mitochondria in the gastrocnemius muscle. On the contrary, a human study showed that when trained males reduced their intake of simple carbohydrates before and during exercise, there were increased signals for mitochondrial biogenesis [40].

The decreased physical performance induced by excessive SSB intake could also be explained through carbohydrate-mediated uric acid metabolism. Simple carbohydrate intake could increase urate synthesis in the kidney [28, 41]. Because of this metabolic alteration, there is a decrease in NO synthesis, leading to endothelial dysfunction, which results in vasoconstriction. Thus, greater vascular constriction leads to lower absorption of oxygen, which directly influences the physical performance [42, 43]. We could not study the uric acid metabolism, so this is another limitation of the study.

### 4.1. Limitations and Future Perspectives

The limitations of the present study were that we did not investigate about mechanisms involved in the decrease in the physical performance, for example, the uric acid metabolism and the heart rate variability in volunteers undergoing in conditions, this variables must be explored in future studies. Other limitation was that were not recorded the food consumption by a 24-hour recording, future studies must do the dietary recall at least 3 times by week during time of experiments.

## 5. Conclusion

The present study shows that only 15 days of excessive SSB intake may negatively modulate biochemical parameters associated with cardiovascular risk. In addition, this overintake can impair the physical performance and cardiovascular responses to physical exercise.

## Data Availability

This manuscript is available at the Institutional Repository of the Federal University of Ouro Preto (http://www.repositorio.ufop.br/handle/123456789/11693) [44].
